# The relationship between glycated hemoglobin level and the stage of periodontitis in individuals without diabetes

**DOI:** 10.1371/journal.pone.0279755

**Published:** 2023-01-06

**Authors:** Arwa Banjar, Rusha Alyafi, Ali AlGhamdi, Mohammad Assaggaf, Ammar Almarghlani, Shaymaa Hassan, Brian Mealey

**Affiliations:** 1 Department of Periodontics, Faculty of Dentistry, King Abdulaziz University, Jeddah, Saudi Arabia; 2 Department of Oral Medicine and Periodontology, Faculty of Dentistry, Cairo University, Cairo, Egypt; 3 Department of Periodontics, University of Texas Health Science Center, San Antonio, Texas, United States of America; Klinikum der Johann Wolfgang Goethe-Universitat Frankfurt Klinik fur Nuklearmedizin, GERMANY

## Abstract

Glycemic control appears to have a significant impact on the relationship between periodontitis and diabetes. The current study aimed to investigate the association between the stage of periodontitis and hemoglobin A1c (HbA1c) levels in patients considered to be normoglycemic. A total of 135 patients (100 females and 35 males) with no history of diabetes were included in the study. The mean age of the participants was 38.4 years old. All patients underwent a full-mouth periodontal examination. Periodontal diagnosis was determined according to the 2017 World Workshop on the Classification of Periodontal Disease. The glycemic state of the patients was assessed using a chair-side HbA1c analyzer. Ninety patients were diagnosed with periodontitis. Higher average HbA1c levels were associated with the different stages of periodontitis (p<0.01). Most of the non-periodontitis patients were in the non-diabetes group (67%), while most of the periodontitis patients were in the undiagnosed pre-diabetes group (47% of the stages I and II group, and 44% of the stages III and IV groups) (p<0.001). Periodontitis was found to be significantly associated with elevated glycated hemoglobin levels in patients not previously diagnosed with diabetes, and the elevation in HbA1c levels was more evident in patients with stage III and IV periodontitis.

## Introduction

Diabetes is a metabolic disease characterized by sustained hyperglycemia due to defects in insulin secretion or uptake. Global reports have shown an alarming increase in the number of people affected by diabetes worldwide [1]. According to the International Diabetes Federation, diabetes prevalence has increased by 62% between 2009 and 2019 [2]. It has also been estimated that half of people with diabetes remain undiagnosed, during which micro- and macrovascular complications may already develop [2].

Periodontitis is a highly prevalent inflammatory condition that results in the destruction of connective tissue attachment and alveolar bone supporting teeth. Periodontal diseases and diabetes are closely associated with chronic conditions. Periodontitis is a well-established comorbidity of diabetes [3]. Epidemiological studies have found that people with diabetes are approximately 3–4 times more likely to experience a severe and progressive form of periodontitis [4, 5]. A recent meta-analysis comparing periodontal health of diabetic patients and healthy individuals concluded that the prevalence of periodontitis is 1.7 times greater in those with diabetes [6]. The state of hyperglycemia elicits an exaggerated immune-inflammatory response to periodontal pathogens in the subgingival environment leading to a severe form of periodontal destruction [7]. Many authors have found that this relationship is independently bidirectional [8, 9]. Previous research has explored the pathophysiology by which periodontal disease affects diabetes and vice versa [8, 10]. Persistent periodontal infection may negatively impact the metabolic state of diabetic patients. Periodontitis is an inflammatory disease, and inflammation plays a major role in diabetes. Polak et al. [11] summarized the possible mechanisms by which periodontitis affects glycemic control. They suggested that the persistence of local inflammation in cases of untreated periodontitis leads to ample production of inflammatory mediators such as tumor necrosis factor, Interlukin-6, C-reactive protein, and oxygen radicals. These inflammatory mediators, together with the bacterial antigens sourced from the plaque microbial biofilm, enter the bloodstream and upregulate the systemic inflammatory state. Consequently, insulin signaling is negatively affected, and insulin resistance is increased [11].

The exact biological mechanisms associated with diabetes and periodontitis are not entirely understood. However, the level of glycemic control appears to be a fundamental factor linking both diseases in a dose-dependent manner. The severity of periodontitis in patients with diabetes is greatly influenced by their metabolic state [12, 13]. In a prospective clinical study, Costa et al. [14] found that elevated hemoglobin A1c (HbA1c) level is significantly associated with the progression of periodontitis and tooth loss. Similarly, Demmer et al. [15] detected up to a threefold increase in the probability of tooth loss in patients with severe hyperglycemia. In contrast, few studies have explored the relationship between periodontitis and glycosylated hemoglobin in patients not diagnosed with diabetes. A pilot study by Wolff et al. [16] reported a higher mean HbA1c level in periodontitis patients than that in non-periodontitis controls after adjusting for several variables. Although the difference in HbA1c levels between the two groups was small (0.12%), it was statistically significant. A large population-based study also found that the presence of chronic periodontal infection at baseline was positively correlated with changes in HbA1c levels over time [17]. The evidence presented thus far suggests that the HbA1c test might be utilized as an objective measure to predict diabetes risk in periodontitis patients or to predict the severity and progression of periodontitis in people with diabetes.

The periodontitis classification was updated in 2017 to align with the current scientific evidence on periodontal disease pathophysiology and risk factors. A grade part that addressed individual variability in disease progression was added. Additionally, well-established risk factors for periodontitis, such as glycemic control and smoking, were used as grade modifiers [18]. Similar to previous periodontal disease classification systems, diabetes-associated periodontitis is not considered a separate disease entity because of the lack of evidence supporting the unique pathophysiology linking diabetes and periodontitis [19]. Since the classification was recently changed, previously explored association themes between periodontitis severity and its effect on diabetes should be revisited and reshaped. The goals of this study were: (1) to investigate whether the level of dysglycemia increases with increasing severity of periodontitis (assessed as 2018 EFP/AAP periodontitis stages) and vice versa.

## Materials and methods

This cross-sectional study was performed between 2017 and 2019 and was approved by the research ethics committee of King Abdulaziz University, Faculty of Dentistry (study number 081-10-17). This study was conducted in accordance with the Helsinki Declaration of 1975, as revised in 2013. All participants in the study signed a written informed consent. The study population was recruited from patients attending undergraduate dental clinics, graduate periodontal clinics, and periodontics specialty clinics at the School of Dentistry, King Abdulaziz University. The inclusion criteria were as follows: 1) age above 18 years; and 2) no previous diagnosis of diabetes or prediabetes. Exclusion criteria were as follows: 1) use of hypoglycemic medications; 2) current long-term use of corticosteroids; 3) renal insufficiency; 4) pregnant women; 5) patients with human immunodeficiency virus infection; and 6) antibiotic use within the last 6 months or need for prophylactic antibiotics before dental procedures.

### Study groups and sampling

Following a comprehensive periodontal examination, eligible patients were divided based on their periodontal diagnosis into three categories: 1) no periodontitis, 2) periodontitis stages I and II, and 3) periodontitis stages III and IV. An equal number of participants were randomly selected from each category.

### Sample size calculation

Sample size was calculated using G*Power version 3.1.9.4 to detect an effect size of 0.36 with a degree of freedom of 2, representing a 0.2 differential in HbA1c between the three equally sized periodontitis groups (5.5%, 5.7%, and 5.9%, respectively), by assuming an overall mean (standard deviation [SD]) HbA1c of 5.7% (0.45) in a group of 38 among non-diabetic individuals [20]. The statistical power was set to 0.9, and the type 1 error was set to 0.05. The total sample size was 102 (34 participants in each periodontitis group); however, the sample size was increased to 45 patients per group for higher statistical power.

### Periodontal assessment

Patients underwent a full-mouth periodontal examination performed by two periodontists (Arwa Banjar and Rusha ALyafi). The examination included measurements of probing depth and gingival margin position using a UNC 15 periodontal probe. Measurements were taken at six sites per tooth for all teeth in the mouth, except for the third molars. The clinical attachment level (CAL) was calculated by adding the probing depth (PD) reading to the gingival margin level when recession was present, and by subtracting the gingival margin level from the PD reading when the gingival margin was coronal to the cemento-enamel junction. Alveolar bone loss was determined in the molar and premolar teeth on horizontal bitewings radiographs, and in the anterior teeth on periapical radiographs. All radiographs were taken and assessed before periodontal examination.

Periodontitis was established according to the 2018 EFP/AAP Periodontitis Classification for Periodontal Diseases and Conditions [21]. Patients were considered to have periodontitis when there were at least two non-adjacent interproximal sites with CAL >2 mm. The site with the greatest loss of clinical attachment was used to establish the stage of periodontitis. When other complexity factors were present, the stage shifted to a higher degree. Periodontitis stage was categorized as follows:

- Stage 1: CAL was 1–2 mm, bone loss was less than 15%, all PD were 4 mm or less, and no tooth loss due to periodontitis.
- Stage 2: CAL was 3–4 mm, bone loss was less than 15–33%, all PD were 5 mm or less, and no tooth loss due to periodontitis.
- Stage 3: CAL was ≥5 mm, bone loss beyond the middle of the root, and loss of no more than four teeth due to periodontitis. The stage of periodontitis was shifted to stage 3 in the presence of any of the following: PD measurements >5 mm, vertical bone loss >3 mm, and grade II or III furcation involvement.
- Stage 4: CAL was ≥5 mm, bone loss beyond the middle of the root, and loss of more than four teeth due to periodontitis. The stage of periodontitis was shifted to stage 4 in the presence of any of the following: secondary occlusal trauma with grade 2 mobility, bite collapse, less than 20 teeth remaining, and the patients’ need for full mouth rehabilitation.

### Assessment of HbA1C

The glycemic state of the subjects was determined using the chair-side HbA1C, which was defined according to the American Diabetes Association criteria [22]. The HemoCue HbA1c 501 (Angelholm, Sweden) analyzer was certified according to the International Federation of Clinical Chemistry and Laboratory Medicine and the National Glycohemoglobin Standardization Program. First, the test cartridge was placed in the analyzer, and a 4 μL finger-prick capillary blood sample was obtained. Blood samples were collected from the sampling area of the reagent pack, which was then inserted into the cartridge test in the analyzer. The percentage of HbA1c levels for each patient was available within 5 min. Single-use check-up cartridges were used for quality control every month before the samples were tested, or on suspicion of inaccurate results.

Despite that none of the patients had been previously diagnosed with diabetes, patients were recognized in the diabetes category if the HbA1c percentage was 6.5% or higher; in the prediabetes category if the HbA1c was between 5.7% and 6.4%; and in the non-diabetes category if the HbA1c was less than 5.7%.

### Body mass index (BMI) assessment

The BMI was calculated using the height and weight measurements, and patients were categorized as underweight if the BMI <18.5 kg/m^2^; healthy weight if the BMI is 18.5 to 24.9 kg/m^2^; overweight if the BMI is 25 to 30 kg/m^2^; or obese if the BMI >30 kg/m^2^.

### Other data

All participants completed a questionnaire that comprised of questions about the patient’s age, sex, level of education, family history of diabetes, signs and symptoms of diabetes, history of gestational diabetes in women, smoking status, history of hypertension, and dyslipidemia.

### Statistical analysis

The mean HbA1c level and SD were calculated based on the characteristics of the study population. The inter-rater reliability for probing depth measurement was analyzed using interclass correlation (ICC) coefficient Model 3 (Two-Way Mixed Model), with calculation of the 95% confidence interval (95%CI). Good inter-rater reliability was considered for an ICC value ≥0.75 (REF). Descriptive statistics were also calculated for the study participants according to the category of diabetes. Chi-square tests were used to compare categorical variables, while t-tests and analysis of variance were used to compare continuous variables. Linear regression models were used to estimate the mean change in HbA1c compared to the periodontitis stage I/II group and stage III/IV group to the non-periodontitis group (reference). The model was adjusted for confounding variables including age, sex, BMI, smoking, level of education, and family history of diabetes. The power of the analysis, α = 0.5, was calculated to be 0.95. Statistical analysis was performed using SAS statistical software (version 9.4; SAS Institute, Cary, NC, USA).

## Results

The study included 135 adults aged between 21 and 73 years. Demographic data from the periodontal diagnosis are presented in Table 1. The total number of subjects diagnosed with periodontitis was 90 (50% stage I/II and 50% stage III/IV). A comparison between periodontally healthy patients and patients with any stage of periodontitis revealed no significant difference in sex distribution between the groups. The mean age of the patients with periodontitis was significantly higher than that of the patients without periodontitis (p<0.001). Regarding educational level, patients with periodontitis were more likely to present with low educational attainment (p<0.001). Smoking history did not differ significantly between the periodontitis and non-periodontitis groups. Conversely, the BMI was higher in periodontitis patients, and periodontitis patients were more likely to have a family history of diabetes. Table 1 shows a significantly greater prevalence of diabetes in patients with periodontitis.

**Table 1:** Characteristics of the study population by periodontitis diagnosis.

Characteristics	Total (N = 135)	No periodontitis (N = 45)	Periodontitis Stages I & II (N = 45)	Periodontitis Stages III & IV (N = 45)	P-value
**Sex**					0.48
Female	100 (74%)	33 (33%)	31 (31%)	36 (36%)	
Male	35 (26%)	12 (34%)	14 (40%)	9 (26%)	
**BMI** ^*^					<0.001
≤24.9	47 (36%)	25 (53%)	13 (28%)	9 (19%)	
25–29.9	43 (33%)	14 (33%)	16 (37%)	13 (30%)	
≥30	40 (32%)	4 (10%)	16 (40%)	20 (50%)	
**Smoking**					0.25
Former	11 (8%)	3 (27%)	4 (36%)	4 (36%)	
Current	20 (15%)	7 (35%)	9 (45%)	4 (20%)	
**Education** ^*^					<0.001
None	9 (7%)	2 (22%)	3 (33%)	4 (44%)	
Elementary	16 (12%)	2 (13%)	6 (38%)	8 (50%)	
Intermediate	13 (9%)	0 (0%)	4 (31%)	9 (69%)	
Secondary	37 (27%)	10 (27%)	17 (46%)	10 (27%)	
College or higher	50 (38%)	29 (58%)	15 (30%)	6 (12%)	
**Family history of diabetes**					0.3
No	59 (44%)	27 (46%)	16 (27%)	16 (27%)	
Yes	76 (56%)	18 (24%)	29 (38%)	29 (38%)	
**Age**					<0.001
Mean (SD)	38.4 (11.1)	29.7 (6.2)	40.8 (10.4)	44.7 (10.0)	
**Diabetes Dx**					<0.001
No diabetes	58 (43%)	30 (52%)	16 (28%)	12 (21%)	
Pre-diabetes	55 (41%)	14 (25%)	21 (38%)	20 (36%)	
Diabetes	22 (16%)	1 (5%)	8 (36%)	13 (59%)	

N do not add up to 135 due to missing data.

BMI, body mass index; SD, standard deviation; Dx, diagnosis.

Chi-squared tests for categorical variables, and ANOVA for continuous (age) variable.

The bivariate correlation between the probing depth measurements of the two examiners was 0.808. The inter-rater reliability analysis showed an ICC value of 0.806 (95%CI = 0.702–0.877), indicating a good inter-rater reliability. The standard error of mean was 0.888, indicating that 68% of the repeat measures are likely to fall within ± 0.888 of the true probing depth (results not presented in tables).

**Fig 1:**
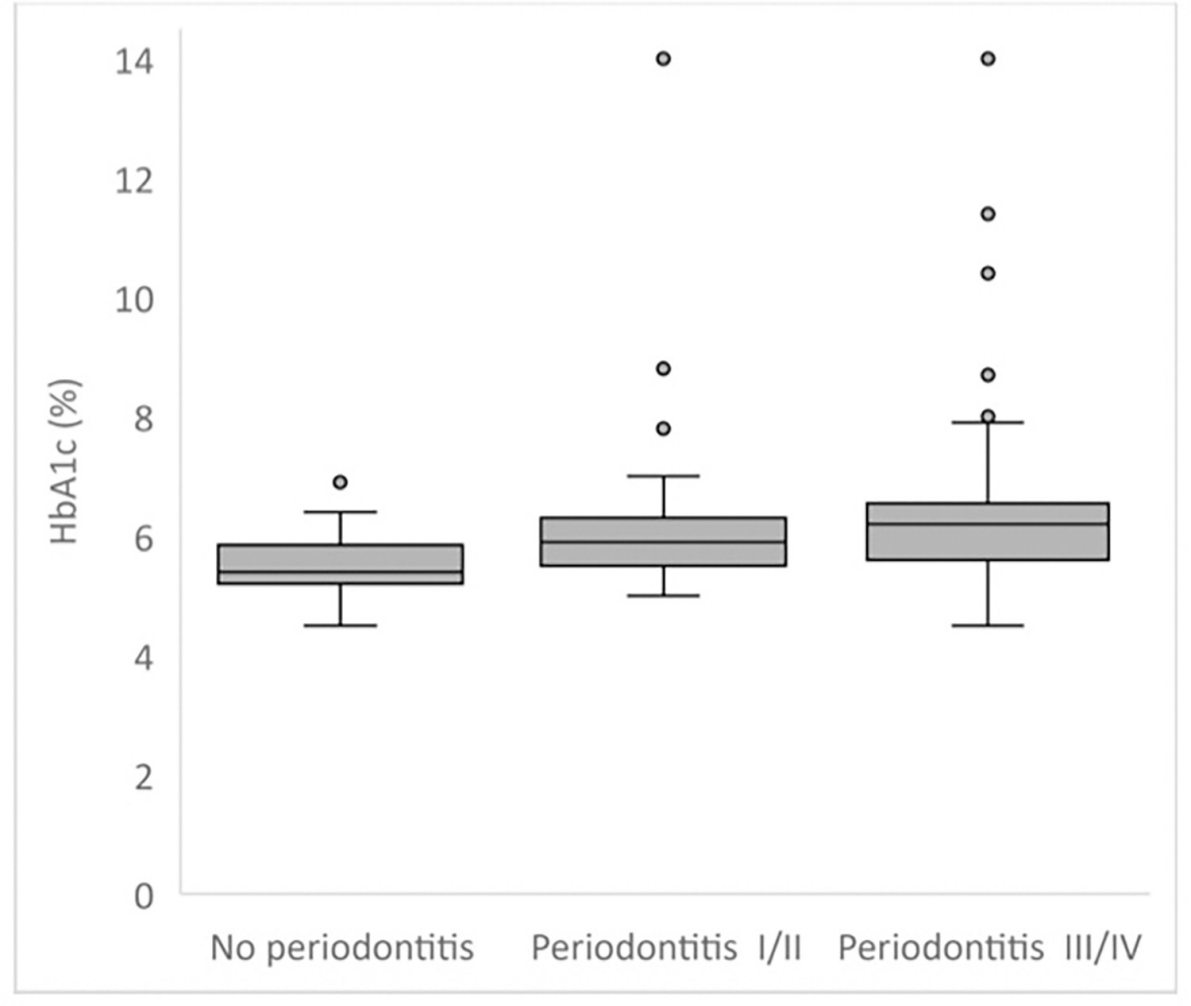
Distribution of HbA1c level in periodontally healthy patients and patients with periodontitis (p<0.01). HbA1c, hemoglobin A1c.

The mean HbA1c values for the study population according to the main exposure and covariate categories are presented in Table 2. Higher average HbA1c levels were associated with different stages of periodontitis (p<0.01). Compared with the healthy weight group, overweight and obese individuals had higher HbA1c levels (p = 0.04). Out of the 135 subjects included, most of the non-periodontitis individuals (67%) were in the non-diabetes group, while most of the periodontitis individuals (47% stage I/II and 44% stage III/IV) were in the previously undiagnosed pre-diabetes group (p<0.001) (Table 3). Periodontitis stages III and IV had the highest proportion of previously undiagnosed diabetes (29% out of the 45 subjects diagnosed with periodontitis stages III and IV). Those with pre-diabetes and diabetes tended to be older (p<0.001). The distribution of HbA1c levels in periodontally healthy patients and patients with periodontitis is shown in Fig 1. More patients with HbA1c levels greater than 6.4% were found in patients with stage III/IV periodontitis (p<0.01).

**Table 2:** Mean HbA1c and SD by periodontal diagnosis and study covariates.

Characteristic	N	Mean HbA1c (SD)	P-value
**Periodontal diagnosis**			<0.01
Periodontal health	45	5.52 (0.49)	
Periodontitis stages I & II	45	6.17 (1.41)	
Periodontitis stages III & IV	45	6.52 (1.71)	
**Sex**			0.94
Female	100	6.07 (1.35)	
Male	35	6.08 (1.43)	
**BMI** ^*^			0.04
≤24.9	47	5.67 (0.69)	
25–29.9	43	6.16 (1.38)	
≥30	40	6.49 (1.84)	
**Smoking**			0.22
Former	11	6.01 (0.96)	
Current	20	5.79 (0.59)	
**Education** ^*^			0.07
None	9	6.08 (0.40)	
Elementary	16	6.86 (2.28)	
Intermediate	13	6.38 (1.22)	
Secondary	37	6.04 (1.50)	
College or higher	50	5.70 (0.69)	
**Family history of diabetes**			0.79
Yes	76	6.04 (1.35)	
No	59	6.10 (1.39)	

N do not add up to 135 due to missing data.

ANOVA and t-tests.

HbA1c, hemoglobin A1c; SD, standard deviation; BMI, body mass index.

**Table 3:** Characteristics of the study population by diabetes diagnosis.

Characteristic	No diabetes (N = 58)	Pre-diabetes (N = 55)	Diabetes (N = 22)	P-value
**Periodontal diagnosis**				<0.001
Periodontal health	30 (67%)	14 (31%)	1 (2%)	
Periodontitis stages I & II	16 (36%)	21 (47%)	8 (18%)	
Periodontitis stages III & IV	12 (27%)	20 (44%)	13 (29%)	
**Sex**				0.65
Female	41 (41%)	43 (43%)	16 (16%)	
Male	17 (49%)	12 (34%)	6 (17%)	
**BMI** ^*^				0.09
≤24.9	25 (53%)	18 (38%)	4 (8%)	
25–29.9	15 (35%)	21 (49%)	7 (16%)	
≥30	16 (40%)	13 (33%)	11 (28%)	
**Smoking**				0.26
Former	5 (45%)	4 (36%)	2 (18%)	
Current	10 (50%)	9 (45%)	1 (5%)	
**Education** ^*^				0.06
None	2 (22%)	6 (67%)	1 (11%)	
Elementary	4 (25%)	5 (31%)	7 (44%)	
Intermediate	4 (31%)	6 (46%)	3 (23%)	
Secondary	17 (46%)	15 (41%)	5 (14%)	
College or higher	28 (56%)	18 (36%)	4 (8%)	
**Family history of diabetes**				0.93
Yes	32 (42%)	32 (42%)	12 (16%)	
No	26 (44%)	23 (39%)	10 (17%)	
**Age**				<0.001
Mean (SD)	34.2 (9.6)	40.2 (11.0)	45.0 (10.6)	
**HbA1c**				<0.001
Mean (SD)	5.30 (2.9)	6.04 (0.23)	8.18 (2.28)	

*N do not add up to 135 due to missing data.

BMI, body mass index; SD, standard deviation; HbA1c, hemoglobin A1c.

Chi-squared tests for categorical variables, and ANOVA for continuous (age and HbA1c) variables.

Table 4 presents the results of the linear regression analysis. In the crude model, those with periodontitis stages I and II had HbA1c that was, on average, 0.65% (absolute value) higher than those without periodontitis (95% confidence interval [CI]: 0.11–1.20, p = 0.02), while periodontitis stages III and IV individuals had HbA1c that was on average 1.00% (absolute value) higher than those without periodontitis (95% CI: 0.46–1.55, p<0.001). After adjusting for age, the association was attenuated but remained statistically significant in the periodontitis stage III/IV group but lost its significance in the periodontitis stage I/II group. In the fully adjusted model, those with periodontitis stage III/IV had HbA1c that was 0.85 on average higher than those without periodontitis (95% CI: 0.14–1.56, p = 0.02), while periodontitis stage I/II had HbA1c that was on average 0.56, higher than those without periodontitis (95% CI: -0.09–1.2, p = 0.09).

**Table 4:** Mean increase in HbA1c by periodontal diagnosis (linear regression).

	Mean increase in HbA1c (95% CI)	P-Value
**Model 1**		
No Periodontitis (Reference)		
Periodontitis stages I & II	0.65 (0.11–1.20)	0.02*
Periodontitis stages III & IV	1.00 (0.46–1.55)	<0.001*
**Model 2**		
No Periodontitis (Reference)		
Periodontitis stages I & II	0.57 (-0.05–1.19)	0.07
Periodontitis stages III & IV	0.90 (0.23–1.56)	<0.01
**Model 3**		
No Periodontitis (Reference)		
Periodontitis stages I & II	0.56 (-0.09–1.20)	0.09
Periodontitis stages III & IV	0.85 (0.14–1.56)	0.02*

Statistically significant.

Model 1: Unadjusted. Model 2: Adjusted for age, and sex. Model 3: Adjusted for age, sex, BMI, smoking, education, and family history of diabetes. Hb1Ac, hemoglobin A1c; CI, confidence interval.

## Discussion

The relationship between periodontitis and diabetes has been an area of extensive research for several decades. The present study supports the literature that periodontitis has a negative effect on glycemic control. The results of the present study indicate that patients diagnosed with periodontitis are more prone to have higher mean HbA1c levels than periodontally healthy individuals. Additionally, patients with periodontitis were more likely to have undiagnosed prediabetes or diabetes than those without periodontitis. This analysis of 135 patients (90 with periodontitis and 45 without periodontitis) revealed that 23.3% (21/90) of the periodontitis group had an HbA1c level consistent with the diagnosis of diabetes, compared to only 2% in the non-periodontitis group. Furthermore, 45.5% (41/90) of periodontitis patients had previously undiagnosed pre-diabetes compared to 31.1% (14/45) of patients without periodontitis. The influence of periodontitis on mean HbA1c level was more evident in patients diagnosed with stage III/IV periodontitis.

In the current study, the definition of periodontitis cases and data categorization for periodontitis stages were based on the consensus of the 2017 World Workshop on the classification of periodontal diseases and peri-implant conditions [21]. Unlike previous classification schemes, the 2018 classification addressed the effect of glycemic control on periodontitis by adopting a two-dimensional approach for periodontitis diagnosis. The stage section describes the severity and complexity of the management of periodontitis, while the grade section assesses individuals’ susceptibility to disease progression based on well-established risk factors [18]. Similar to previous periodontal disease classification systems, diabetes-associated periodontitis is not considered as a separate disease entity because of the lack of evidence to support a unique pathophysiology for patients with diabetes and periodontitis [19]. Nevertheless, diabetes, with HbA1c level as a reference range, was set as a grade modifier and risk assessor [21]. To the best of our knowledge, this study is the first to explore the association between the stages of periodontitis and HbA1c levels. Since the classification of periodontitis was recently updated, previously explored association themes between periodontitis severity and its effect on diabetes should be revisited and reshaped.

In 2011, the World Health Organization approved HbA1c as a test for the diagnosis of diabetes [23]. In the current study, HbA1c levels were measured using a commercially available point-of-care system (HemoCue HbA1c 501 analyzer). The device is convenient to use and provides HbA1c results within a few minutes, allowing for instant feedback. The analytical performance of the device used in this study was evaluated in a primary-care setting. The study reported 6% bias and a total coefficient of variation of less than 2% for more than 96% of the results, meeting the National Glycohemoglobin Standardization Program requirements [24].

Consistent with the findings of the current study, Saito et al. [25] demonstrated that in patients without diabetes, more severe forms of periodontitis are associated with impaired glucose tolerance. In this study, the change in HbA1c levels was evaluated retrospectively for 10 years. It was observed that for each millimeter increase in probing depth measurements, the HbA1c level increased by 0.13% [25]. Moreover, Demmer et al. [26] reported that patients with intermediate levels of periodontal disease exhibited a twofold increased risk of diabetes. In a longitudinal study following a non-diabetic population over 5 years, it was shown that severe forms of periodontitis influence HbA1c levels and can be used as a predictor for worsening of HbA1c levels [17]. A recent systematic review by Graziani et al. [27] revealed that glycemic control is compromised in non-diabetic patients with periodontitis. This was demonstrated by higher levels of HbA1c, oral glucose tolerance test results, and fasting blood glucose [27]. In contrast to most findings reported in the literature, one longitudinal study by Kebede et al. [28] failed to find any association between periodontitis and diabetes incidence over an 11 years follow up period. Their analysis demonstrated no potential correlation between changes in HbA1c levels and the baseline periodontal status. The authors have discussed two explanations for this contradictory result. The first was survivor bias, as most periodontitis subjects with a high susceptibility to diabetes were less likely to complete the study. Second, there was a lack of inflammatory progression since participants with incident diabetes/prediabetes were more likely to present with central obesity at baseline [28].

The results of the current study indicate that many of the patients previously thought to have normal glucose dynamics and diagnosed with periodontitis were in the prediabetes category based on their HbA1c levels. In line with this finding, a recent cross-sectional study by Isola et al. [29] found that almost one third of the periodontitis group had HbA1c levels within the range of prediabetes values. It has been previously reported that individuals with prediabetes are more likely to develop diabetes, retinopathy, and cardiovascular disease [30, 31]. One longitudinal study in American Indian population established the utility of HbA1c to predict the incidence of diabetes. The analysis revealed that the incidence of diabetes was higher in children with prediabetes than in those without [32].

This study was based on a sample obtained from a single university dental hospital and cannot be extrapolated to populations with different sociodemographic or environmental backgrounds. In addition, since the data were collected cross-sectionally, it was not possible to determine whether the incidence and progression of periodontal disease preceded the elevation in HbA1c levels. Further longitudinal studies are needed to reveal and specify causality between the different stages of periodontitis and HbA1c levels. Additionally, prospective monitoring of a population with respect to the effect of the stage of periodontitis on HbA1c levels would provide insight into the temporal relationship between periodontitis stages and HbA1c levels.

The observations in the current study suggest that periodontitis patients, particularly those with advanced stages of periodontal disease, may be targeted for opportunistic screening for diabetes. Our suggestion is supported by findings from a previous case control study in a Danish cohort by Holm et al. [33], where chairside HbA1c test was used to screen chronic periodontitis patients with undiagnosed diabetes and revealed that the proportion of patients with undiagnosed dysglycemia was higher in the periodontitis group (32.7%) compared to the control group (17.4%). The reported odds ratio of having undiagnosed diabetes in the study was 2.3 in periodontitis patients compared to that in periodontally healthy patients [33].

Diabetes complications impose a substantial financial burden on people worldwide. The cost of diabetes has been estimated to increase significantly by 2030 [2]. Dental offices have been studied as potential places for opportunistic screening for diabetes [34]. It has been reported that approximately 70% of adults in the U.S visit their dentists at least once a year. Many of them do not see their physicians regularly [35]. Involving dentists in screening for common medical conditions may improve medical diagnoses and reduce healthcare costs. Studies evaluating dentists’ willingness to perform medical screening have revealed that dentists believe in their vital role in screening for medical conditions in their offices [36, 37]. A survey assessing dentists attitudes toward chairside medical screening reported that dentists are willing to use tests that generate immediate results to be discussed with the patient during the dental visit [37]. Further large-scale studies are needed to determine the cost-effectiveness of this patient-centered approach.

## Conclusion

Patients with periodontitis are more likely to present with undiagnosed dysglycemia than periodontally healthy patients. Periodontitis was significantly associated with elevated glycated hemoglobin levels in patients not previously diagnosed with diabetes. The association between mean HbA1c level and periodontal disease was more evident in patients diagnosed with stage III/IV periodontitis.

## Supporting information

S1 File (XLSX)
